# Analysis of Factors Influencing Polish Students’ Opinions on Euthanasia

**DOI:** 10.3390/ijerph19095019

**Published:** 2022-04-20

**Authors:** Iga Stokłosa, Maciej Stokłosa, Gniewko Więckiewicz, Mateusz Porwolik, Maciej Bugajski, Wilhelm Masarczyk, Tomasz Męcik-Kronenberg, Magdalena Piegza, Robert Pudlo, Piotr Gorczyca

**Affiliations:** 1 Department and Clinic of Psychiatry, Medical University of Silesia, 42-612 Tarnowskie Gory, Poland; maciej.piotr.stoklosa@gmail.com (M.S.); gniewkowieckiewicz@gmail.com (G.W.); wil.m@poczta.fm (W.M.); mpiegza@sum.edu.pl (M.P.); rpudlo@sum.edu.pl (R.P.); pgorczyca@sum.edu.pl (P.G.); 2 Department of Ophtalmology, Medical University of Silesia, 40-514 Katowice, Poland; pporwolik@gmail.com; 3 National Research Institute of Oncology, State Research Institute, 31-115 Krakow, Poland; maciek.bugajski@gmail.com; 4 Department of Pathomorphology, Medical Univeristy of Silesia, 41-800 Zabrze, Poland; patolog@interia.pl

**Keywords:** death, ethics, euthanasia, public health, students

## Abstract

Due to the continuous development of palliative care and methods of pain relief in the last moments of patients’ lives, we are faced with the question of how long we should take measures to delay inevitable death for, without providing prolonged therapy against the patient’s will. For terminally ill and people experiencing unimaginable suffering, euthanasia is considered as one of the possible options for a dignified farewell. The aim of the study was to determine the views on euthanasia in a group of students from Polish universities. Questionnaire responses were analyzed for 9686 students (79.9% of women and 20.1% of men) aged 18–35 years. Respondents were asked to complete their own questionnaire on demographic data and attitudes toward euthanasia in the case of severe terminal illness or paralysis preventing independent living. Euthanasia was significantly more acceptable among women (85% vs. 75%, *p* < 0.0001; 69% vs. 62%, *p* < 0.0001) and non-believers [98% vs. 97% (denominations other than Christian), 84% (other forms of Christianity), 69% (Roman Catholic); *p* < 0.0001] in every case considered. Religious affiliation was the factor that most influenced attitudes toward euthanasia. Among the other elements influencing attitudes toward euthanasia, residence and field of study were distinguished. Considering the large sample size and specific conclusions, the article should be an important argument in the discussion on euthanasia.

## 1. Introduction

One of the most ethically controversial issues discussed by the public in many countries, not only from a medical but also from a moral point of view, is euthanasia. By definition, euthanasia is a procedure that aims to end the life of a severely chronically ill person in a painless way when the person experiences unimaginable suffering [1]. This process is divided into three forms: active euthanasia, passive euthanasia, and assisted suicide. Passive euthanasia is the failure to continue to support the patient’s life, while active euthanasia is the taking of measures aimed at ending a person’s life under the influence of compassion for them and at their expressed request [2]. Assisted suicide is a process in which a physician facilitates a patient’s death by providing the patient with the necessary resources or information to enable the patient to commit the death act [3]. Euthanasia means providing the patient with means to commit suicide, while all activities aimed at taking one’s own life are carried out by the patient [4]. Currently (2022), euthanasia is legalized in Europe in Belgium, the Netherlands, and Luxembourg, and, according to recent reports, this procedure is recognized as legal in Spain [5,6]. The remaining countries in the world where euthanasia is available are Canada and Colombia [7]. Depending on the country, the reasons for euthanasia vary, but most often this procedure is possible in the case of a serious and incurable disease, both physical and psychological, which causes unimaginable suffering that cannot be alleviated [8]. People who support the possibility of euthanasia state as their main argument that they want to ensure that suffering and terminally ill people have the right to die with dignity. In addition, many patients in a vegetative state do not want to be a burden to their family members, and demand the “right to die”. Another argument of the proponents of this procedure is the possibility of using healthy organs from patients who choose euthanasia, and transferring them to people waiting for transplantation [8]. Opponents of euthanasia believe that this practice can eliminate terminally ill people from society, while the current development of palliative care allows suffering patients to relieve their pain and improve the comfort of their functioning. In addition, opponents of euthanasia mention as counterarguments the fear of possible abuse, as well as the violation and constant shifting of the ethical boundaries of the current criteria for approval of euthanasia [9,10]. Euthanasia remains an issue that, unlike other moral dilemmas such as abortion and in vitro fertilization (IVF), is forbidden in all major religious cultures of the world [11]. In recent years, there have been frequent discussions about the indications for the possibility of euthanasia in children suffering from terminal illnesses [12]. Now, 20 years after the legalization of euthanasia in the Benelux countries, there is also a growing percentage of people who choose to receive help to end their lives, and are struggling with severe mental disorders such as dementia, major depression, bipolar disorder, and conversion disorder [13,14]. Many mental disorders, including depression, remain controversial as a criterion for assisted suicide. In these cases, the ability to give informed consent by the patient remains a topic of discussion, especially when it is accompanied by the deterioration of cognitive function [15]. The same can be said about cognitive disorders and dementia in general. From the available data, it can be concluded that, in the Netherlands, euthanasia was reported as the cause of death in 2.9% of deaths (2010), while in Belgium, this figure was 4.6% (2013), but these figures are constantly increasing—4.4% in the Netherlands (2017), while in Belgium, the number of reported cases of euthanasia in 2021 increased by 10.39%, as compared to 2020 [16,17,18]. According to the Annual Report, the main cause of euthanasia in countries that have legalized this procedure is cancer, but also many comorbidities and neurological disorders [19]. In addition, it should be noted that reporting euthanasia as a cause of death is a complex and often problematic issue. Therefore, it has been suggested that the number of assisted deaths is sometimes underestimated and that there are actually more of them than the statistical data indicates [20]. The aim of this study was to determine attitudes toward euthanasia among students at Polish universities—people who are also studying in the field of medicine and may in the future come into direct contact with moral dilemmas related to the issue of patient life and death. In our study, we aimed to identify the factors that determine young adults’ attitudes toward euthanasia. In addition, we wanted to draw attention to a topic that has remained a frequently discussed aspect of ending human life in recent years in an ethical and moral context, around which many controversies and questions arise that often remain unanswered. It is important from the perspective of public health to understand the needs and expectations of Polish society regarding this difficult moral and medical dilemma.

## 2. Materials and Methods

### 2.1. Sample Characteristics

A total of 9824 students aged 18–35 years from 40 Polish universities participated in the study. The criteria for inclusion in the study were student status and correct completion of the questionnaire. After a thorough analysis of the collected responses, the inclusion criteria were met by 9686 respondents, whose questionnaires were then subjected to statistical analysis. Among the students, two groups were distinguished—respondents studying in the medical field (medicine and paramedical studies), and students of other scientific fields.

### 2.2. Questionnaire

The questionnaire included questions on sociodemographic data and attitudes toward the aspect of euthanasia (Appendix A). In terms of population data, students indicated their gender, age, field and year of study, and place of residence. A corresponding euthanasia questionnaire allowed respondents to select the “for” or “against” option in two cases involving the circumstances of euthanasia: (1) a serious, incurable illness that causes unimaginable suffering; (2) permanent paralysis of the body that substantially limits the ability to function independently. Respondents were asked whether the method of euthanasia should be legal in a particular case, whether they would choose euthanasia themselves, and also whether they would choose euthanasia for their relatives if they asked for it.

### 2.3. Distribution of the Questionnaire

Students were asked to complete their own questionnaire in Polish, which was prepared by the authors of this paper, and then carefully analyzed by two independent experts in the field of public health and ethics. In order to ensure the greatest possible anonymity and convenience in filling out the questionnaire, and to reach the most diverse and large group of students, it was decided to distribute the survey via the Internet, using Google Forms, Google’s proprietary platform that enables the creation of anonymous questionnaires that are convenient for both creators and participants. A total of 40 Polish universities were randomly selected, and then, using social media, the researchers managed to reach online groups in which students from each university had joined, and in which, with the administrator’s consent, the form was distributed to be filled out and participants informed about the purpose of the study, and the appropriate consents to participate in the study were obtained. In each survey we sent, participants received information about the purpose of the study and were asked for consent to participate in it by selecting the appropriate option in the consent form available to them immediately before going to the appropriate questionnaire. The survey was available to participants from 10 October to 10 November 2018. Initially, the questionnaire was tested on a group of 30 students from the Medical University of Silesia in Katowice. Since the project was conducted online in a manner that prevented the identification of respondents, the study did not require approval from the Bioethics Committee. The study was exploratory in nature, and intended to determine students’ views on euthanasia. The currently described topic of euthanasia is one of the parts of the project on attitudes of Polish university students towards controversial ethical and health issues, for example euthanasia, abortion, and IVF. The first part of the study on attitudes towards abortion was published in the International Journal of Environmental Research and Public Health (IJERPH) in December 2021 [21].

### 2.4. Statistical Analysis

The collected data were analyzed using the STATISTICA 13.3 program (StatSoft, Krakow, Poland). The significance level was set at *p* < 0.01. For comparison of qualitative variables, the chi-square test was used, and for quantitative variables that did not conform to the normal distribution, the Mann–Whitney U test was used to compare two groups. For additional groups, the Kruskal–Wallis test was used. To evaluate the strength of correlation, the Spearman rank test was used.

## 3. Results

The characteristics of the group and the distribution of responses by gender are shown in Table 1.

A total of 9686 respondents (79.9% women and 20.1% men) participated in the study. The age of women in the study was significantly higher than that of men. Women are statistically significantly more supportive of the legality of euthanasia, more often explaining the possibility of euthanasia or the acceptance of euthanasia among their family members compared to male respondents.

The distribution of responses as a function of medical school or other studies is shown in Table 2.

Due to the possibility of direct contact with the topic of euthanasia during education and work, it was decided in the following study to divide the respondents into groups studying medicine and those studying outside the medical field. Medical students were significantly less likely than other majors to support the legality of euthanasia, and would be less likely to provide euthanasia in certain cases queried by the researchers.

The distribution of responses as a function of reported religion is shown in Table 3.

The study also analyzed the influence of religion on attitudes toward euthanasia. Faith is a statistically significant and strong factor determining respondents’ views on euthanasia. Opposition to euthanasia increases according to group affiliation in the following order: (1) non-believers, (2) believers in religions other than Christian, (3) believers in non-Roman Catholic faiths, (4) Roman Catholic.

The distribution of results according to the size of the respondent’s city of origin is shown in Table 4.

The acceptance of euthanasia increases with the size of the respondent’s city of origin.

The distribution of responses as a function of the respondent’s field of study is shown in Table 5.

Significant differences were found between fields of study in attitudes toward euthanasia; in every case analyzed, the differences were statistically significant. The most conducive of euthanasia were social sciences, arts, and natural sciences, while the opposite views were noticed in the field of education, law, and religious subjects. There was a discrepancy between the medical field and paramedical fields (such as emergency services, nursing, medical caregivers), where responses differed by an average of 10%, placing them at two poles of the list. In every case studied, age was an important factor influencing respondents’ views. Age was significantly higher in the groups that were in favor of each analyzed case of euthanasia than in the groups that had a different opinion.

The average age of the respondents depending on the given answer in each analyzed case is shown in Table 6.

## 4. Discussion

Euthanasia continues to be a complex ethical and legal issue. A wide-ranging discussion has consistently argued that legalizing the practice could be a dangerous entry into a downward spiral and the possibility of significant abuse [22]. A review of the scientific literature suggests that in countries that have allowed the possibility of euthanasia, as well as in countries that have decided to legalize so-called assisted suicide, many terminally ill people choose to avail themselves of the opportunity to end their lives with the participation of others [19]. To date, studies have been conducted in many countries to determine the attitudes of certain social groups toward assisted suicide [23,24]. Furthermore, in Poland, an attempt was made to characterize the views on the phenomenon of euthanasia among medical students from two Polish universities (588 respondents), but so far no similar study has been conducted in Poland among such a large group of people who are also not associated with the medical profession, which should be emphasized [25].

The factor determining the views on euthanasia among our respondents is gender. Women are significantly more likely to support the possibility of legalizing euthanasia, both in the case of a terminal, serious illness and in the case of paralysis that prevents independent living. Women are also more liberal about undertaking this procedure for themselves and their loved ones. This finding contrasts with data from the Kuwait study, in which men showed greater tolerance of euthanasia [26]. In a study conducted among health science students in Papua New Guinea, no significant differences in attitudes toward this phenomenon were found between the genders [27].

Age was found to be a factor significantly influencing attitudes toward the possibility of assisted termination of existence. As in other studies conducted worldwide, the tendency was observed that older students are more open to the phenomenon of euthanasia than younger people, who are more rigorous about legalization and possible implementation in the case of themselves or their relatives [28].

Respondents’ place of residence also has a significant influence on their respective attitudes toward euthanasia-both in terms of legalization and whether euthanasia is permitted among relatives or in their own case. Residents of larger cities show greater tolerance on these issues, which is consistent with the views of respondents over 50 years of age in Austria, where greater approval of euthanasia is observed among residents of urban areas [29]. 

Euthanasia is considered in all major religious cultures to interfere with the life of another human being, thereby compromising the sanctity of human life, and is a prohibited practice [30]. Among the respondents of this study, it has also been shown that students who belong to a particular religion denomination do not agree with the legalization of this practice. Students professing the Roman Catholic faith were most strongly opposed to euthanasia. Tolerance of the phenomenon of euthanasia grows accordingly among the group of students who report belonging to other groups of Christianity, and then to other religions. The greatest liberalism in the field of euthanasia is shown by people who identify themselves as non-believers. In a 2015 study, religion was also identified among medical students as one of the factors that strongly influence opposition to activities that may contribute to a faster death of patients, and Muslims in particular were opposed to euthanasia [31]. Similar trends were found in a study conducted in England and Wales, where Muslims and Catholics were strong opponents of euthanasia, while Protestants and non-believers showed more liberal attitudes toward issues related to death [32].

Looking at attitudes toward euthanasia among students in specific fields of study, medical school students were characterized by considerable reluctance to legalize euthanasia and implement it. Similar results were obtained in a study conducted in Germany, which demonstrated that both in 2004, and 12 years later in a 2016 study, only a small proportion of students supported the legalization of euthanasia and assisted suicide, possibly indicating that as future physicians they would not be able to perform such an operation on a patient [33]. Another study that examined the views of future physicians also found that medical students do not support euthanasia because it is contrary to the principles of medicine to care for patients by using the available options of medicine and palliative care and to provide them with a dignified death [34].

Our study concluded that the most cautious groups in terms of attitudes toward euthanasia were students from faculties related to cultural studies, religious studies, education, and law. Researchers in India came to different conclusions. There, it was found that among the specific professional groups in which attitudes toward euthanasia were studied, the proponents of this phenomenon were mainly judges [35]. This discrepancy can be explained by the influence of professional experience and age of judges on the views on euthanasia in the study cited above, considering that in our study we asked students, for example, young adults who had not yet practiced their profession, about their views. On the other hand, students in the humanities, natural sciences, and social sciences were significantly more in favor of euthanasia. This might be related to the fact that people who consider themselves artistically talented are characterized by a greater openness to new experiences and boundary crossing, and their attitudes are nonconformist to a greater extent [36].

## 5. Limitations

This study is exploratory in nature; therefore we could not raise any particular research questions, which could be considered a limit in itself. It was conducted to determine the views of young adults in Poland on the issue of euthanasia, which has become increasingly popular in global forums in recent years, but is still a controversial topic treated as an ethical dilemma. In our study, we included only undergraduate students in the questionnaire, therefore there is no complete evaluation of this phenomenon among non-educated people; through evaluation, this particular group could provide more interesting insights. In addition, the study used an original questionnaire which has not been validated, therefore it lacks psychometric evaluation; however, we found no specific questionnaire on euthanasia, and it seemed important to create a questionnaire regarding the practice that could be used in international research to learn about the views of people from different countries on this issue, and then to unify them and indicate the factors that determine them. However, the large number of respondents, which in itself is of great value, encouraged the authors to publish this manuscript.

## 6. Conclusions

From the presented demographical data, it can be concluded that in every case, women show greater tolerance of euthanasia than men. Age influences respondents’ views on euthanasia—the older respondents are, the greater acceptance of euthanasia. The larger the city in which they live, the greater the favor of euthanasia respondents’ views are. The factor that strongly determines attitudes toward euthanasia is religion—the greatest opposition to euthanasia is expressed by the respondents declaring their affiliation to the Roman Catholic Church, and the smallest is observed among non-believers. Considering education—students of religious studies, pedagogy and law are characterized by more cautious views, unlike students of arts, science, and social sciences. Medical students showed greater opposition to euthanasia than paramedics. 

## Figures and Tables

**Table 1:** Distribution of answers according to the gender of the respondents. All of the data are given in %.

		All	Male	Female	χ 2/Z **	*p* Value
Amount		9686	1947	7739		
%		100	20	80		
Mean Age		23.7 ± 3.6	23.0 ± 2.9	23.9 ± 3.8	8.1 **	<0.0001 **
**Do you think that euthanasia should be allowed in the following cases? Yes:**	**Severe disease**	83	75	85	92.9	<0.0001 *
	**Paralysis**	67	62	69	28.9	<0.0001 *
**Would you undergo euthanasia in the following cases? Yes:**	**Severe disease**	73	62	76	118.9	<0.0001 *
	**Paralysis**	59	49	62	72.1	<0.0001 *
**Would you allow a family member to be euthanized in the following cases? Yes:**	**Severe disease**	72	62	74	71.2	<0.0001 *
	**Paralysis**	52	44	54	36.5	<0.0001 *

* *p* Value for Chi-square test; ** *p* Value or *Z* Value for Mann–Whitney U test.

**Table 2:** Distribution of responses depending on the study of medicine or other studies. All of the data are in %.

		All	Non-Med	Medical	χ2	*p* Value
Amount		9686	8582	1104		
%		100	89	11		
**Do you think that euthanasia should be allowed in the following cases? Yes:**	**Severe disease**	83	84	75	41.7	<0.0001 *
	**Paralysis**	67	69	57	52.6	<0.0001 *
**Would you undergo euthanasia in the following cases? Yes:**	**Severe disease**	73	74	64	37.3	<0.0001 *
	**Paralysis**	59	60	49	36.1	<0.0001 *
**Would you allow a family member to be euthanized in the following cases? Yes**	**Severe disease**	72	72			
	**Paralysis**	52	52			

* *p* Value for Chi-square test; Non-med—non medical.

**Table 3:** Distribution of answers depending on the declared religion.

				Believers				
		All	Non-Believers	Non-Christians	Other Christians	Roman Catholics	χ2	*p* Value
Amount		9686	3647	452	253	5334		
%		100	37.6	4.7	2.6	55.1		
**Do you think that euthanasia should be allowed in the following cases? Yes:**	**Severe disease**	83	98	97	84	69	1217.9	<0.0001 *
	**Paralysis**	67	91	89	67	48	1607.9	<0.0001 *
**Would you undergo euthanasia in the following cases? Yes:**	**Severe disease**	73	95	92	68	55	1349.5	<0.0001 *
	**Paralysis**	59	86	81	59	40	1299.6	<0.0001 *
**Would you allow a family member to be euthanized in the following cases? Yes:**	**Severe disease**	72	96	91	69	52	1288.2	<0.0001 *
	**Paralysis**	52	84	77	53	30	1351.3	<0.0001 *

* *p* Value for Chi-square test.

**Table 4:** Distribution of answers depending on the size of the respondent’s city of origin.

		All	Village	<20k	20–50k	50–100k	100–200k	200–500k	>500k	χ2	*p* Value
Amount		9686	1785	924	929	859	827	1347	3015		
%		100	18	10	10	9	9	14	31		
**Do you think that euthanasia should be allowed in the following cases? Yes:**	**Severe disease**	83	75	81	82	81	85	84	87	97.9	<0.0001 *
	**Paralysis**	67	53	64	63	63	72	71	76	238.1	<0.0001 *
**Would you undergo euthanasia in the following cases? Yes:**	**Severe disease**	73	63	72	73	70	76	73	79	106.1	<0.0001 *
	**Paralysis**	59	48	55	59	56	61	61	67	121	<0.0001 *
**Would you allow a family member to be euthanized in the following cases? Yes:**	**Severe disease**	72	58	68	73	70	76	73	79	146.5	<0.0001 *
	**Paralysis**	52	35	44	50	48	57	58	63	216.8	<0.0001 *

* *p* Value for Chi-square test.

**Table 5:** Distribution of answers depending on the field of study of the respondent.

	Do You Think That Euthanasia Should Be Allowed in the Following Cases? Yes:		Would You Undergo Euthanasia in the Following Cases? Yes:		Would You Allow a Family Member to Be Euthanized in the Following Cases? Yes:	
	Severe Disease	Paralysis	Severe Disease	Paralysis	Severe Disease	Paralysis
**Social Sciences**	94%	82%	87%	76%	84%	70%
**Artistic**	89%	78%	79%	71%	78%	62%
**Natural**	87%	72%	79%	66%	78%	60%
**Medical, non-MD**	85%	65%	75%	59%	74%	56%
**Sport**	85%	71%	80%	67%	76%	44%
**Humanistic**	85%	74%	77%	65%	75%	58%
**Another**	84%	69%	76%	62%	72%	53%
**Agriculture**	84%	62%	65%	57%	73%	37%
**Military**	84%	70%	82%	70%	80%	58%
**All**	83%	67%	73%	59%	72%	52%
**Economics and management**	82%	64%	72%	56%	68%	44%
**Mining and metallurgy**	82%	62%	68%	56%	69%	41%
**University of Technology**	80%	64%	67%	53%	67%	46%
**Strict sciences—non-technical**	80%	62%	68%	53%	67%	45%
**Medical**	75%	57%	64%	49%		
**Education**	72%	53%	61%	49%	55%	33%
**Law**	67%	63%	62%	43%	56%	29%
**Religious**	64%	63%	67%	65%	63%	61%
**χ2**	180.2	208.6	162.2	159.4	132.8	179.6
***p* Value**	<0.0001 *	<0.0001 *	<0.0001 *	<0.0001 *	<0.0001 *	<0.0001 *

* *p* Value for Chi-square test; non-MD—non-medical doctor.

**Table 6:** The average age of the respondents depending on the given answer in relation to the given cases.

		Mean Age		With	*p* Value
		Answers for Each Question			
		Yes	No		
Do you think that euthanasia should be allowed in the following cases? Yes:	Severe disease	23.8	23.4	3.2	0.0012 **
	Paralysis	24	23.2	8.6	<0.0001 **
Would you undergo euthanasia in the following cases? Yes:	Severe disease	23.9	23.3	5.7	<0.0001 **
	Paralysis	24	23.2	8.1	<0.0001 **
Would you allow a family member to be euthanized in the following cases? Yes:	Severe disease	24.2	23.4	7.1	<0.0001 **
	Paralysis	24.4	23.2	10.7	<0.0001 **

** *p* Value for Mann–Whitney U Test.

## Data Availability

Data supporting reported results are available on request from the study team.

## Appendix A

1. Gender
  (a) Male
  (b) Female
2. Year of birth
3. Branch of study
  (a) Agriculture
  (b) Another
  (c) Artistic
  (d) Economics and management
  (e) Education
  (f) Humanistic
  (g) Medical
  (h) Medical, non-medical
  (i) Military
  (j) Mining and metallurgy
  (k) Natural
  (l) Social Sciences
  (m) Sport
  (n) Strict sciences—non-technical
  (o) Technology
4. University
5. Year of study
6. Place of residence
  (a) village
  (b) town of <20k inhabitants
  (c) town of 20–50k inhabitants
  (d) town of 50–100k inhabitants
  (e) town of 100–200k inhabitants
  (f) town of 200–500k inhabitants
  (g) town of >500k inhabitants
7. What is your religion?
8. Are you religiousy active?
  (a) Yes
  (b) No
9. Do you think that euthanasia should be allowed in the following cases?
  (A) serious, incurable illness that causes unimaginable suffering; YES/NO
  (B) permanent paralysis of the body that substantially limits the ability to function independently; YES/NO
10. Would you undergo euthanasia in the following cases?
  (A) serious, incurable illness that causes unimaginable suffering; YES/NO
  (B) permanent paralysis of the body that substantially limits the ability to function independently; YES/NO
11. Would you allow a family member to be euthanized in the following cases?
  (A) serious, incurable illness that causes unimaginable suffering; YES/NO
  (B) permanent paralysis of the body that substantially limits the ability to function independently; YES/NO
